# Development of artificial blood loss and duration of excision score to evaluate surgical difficulty of total laparoscopic anterior resection in rectal cancer

**DOI:** 10.3389/fonc.2023.1067414

**Published:** 2023-03-07

**Authors:** Jingfang Lv, Xu Guan, Ran Wei, Yefeng Yin, Enrui Liu, Zhixun Zhao, Haipeng Chen, Zheng Liu, Zheng Jiang, Xishan Wang

**Affiliations:** Department of Colorectal Surgery, National Cancer Center/National Clinical Research Center for Cancer/Cancer Hospital, Chinese Academy of Medical Sciences and Peking Union Medical College, Beijing, China

**Keywords:** rectal cancer, totally laparoscopic anterior resection, surgical difficulty, BLADE score system, random forest algorithm

## Abstract

**Purpose:**

Total laparoscopic anterior resection (tLAR) has been gradually applied in the treatment of rectal cancer (RC). This study aims to develop a scoring system to predict the surgical difficulty of tLAR.

**Methods:**

RC patients treated with tLAR were collected. The blood loss and duration of excision (BLADE) scoring system was built to assess the surgical difficulty by using restricted cubic spline regression. Multivariate logistic regression was used to evaluate the effect of the BLADE score on postoperative complications. The random forest (RF) algorithm was used to establish a preoperative predictive model for the BLADE score.

**Results:**

A total of 1,994 RC patients were randomly selected for the training set and the test set, and 325 RC patients were identified as the external validation set. The BLADE score, which was built based on the thresholds of blood loss (60 ml) and duration of surgical excision (165 min), was the most important risk factor for postoperative complications. The areas under the curve of the predictive RF model were 0.786 in the training set, 0.640 in the test set, and 0.665 in the external validation set.

**Conclusion:**

This preoperative predictive model for the BLADE score presents clinical feasibility and reliability in identifying the candidates to receive tLAR and in making surgical plans for RC patients.

## Introduction

The fast development of laparoscopic surgery indicated great progress in the treatment of colorectal disease in past decades. Substantial evidence suggests that laparoscopic anterior resection (LAR) benefits rectal cancer (RC) patients through a high-definition surgical field, bleeding reduction, early recovery of bowel function, and short hospital stay (1–3). However, conventional LAR requires an abdominal incision for specimen extraction and digestive reconstruction. Despite the incision of LAR being smaller than it is in open surgery, it still causes incisional infection, postoperative pain, and incisional hernia, which could reduce the advantages of minimally invasive laparoscopic surgery (4–6). The introduction of total LAR (tLAR) with intracorporeal anastomosis and natural orifice specimen extraction (NOSE) has led to improvement of short-term outcomes caused by incision (7–9) and has comparable 3-year disease-free and overall survival with those in conventional laparoscopy (10), which has therefore inspired further exploration and popularization of tLAR in the treatment of RC (11–15). However, tLAR is challenged by complicated surgical procedures and high surgical difficulty of intracorporeal anastomosis, as well as potential concerns regarding intraperitoneal contamination and dissemination of tumor cells (11, 16–19).

Scoring systems of surgical difficulty not only help to identify patients with a high risk of postoperative complication and poor prognosis but also help surgeons to select appropriate cases and make surgical plans. Although the predictors of the difficulty of anterior resection have been identified (20–22), no scoring systems have been developed for tLAR. Here, we performed this study with the aims of a) developing a simple clinical tool named blood loss and duration of excision (BLADE) scoring system to evaluate the surgical difficulty of tLAR, b) assessing the effect of the BLADE score on short-term outcomes for RC patients undergoing tLAR, and c) using preoperative variables to establish the predictive model for the BLADE score based on machine learning algorithms.

## Materials and methods

### Study population

**Figure 1:**
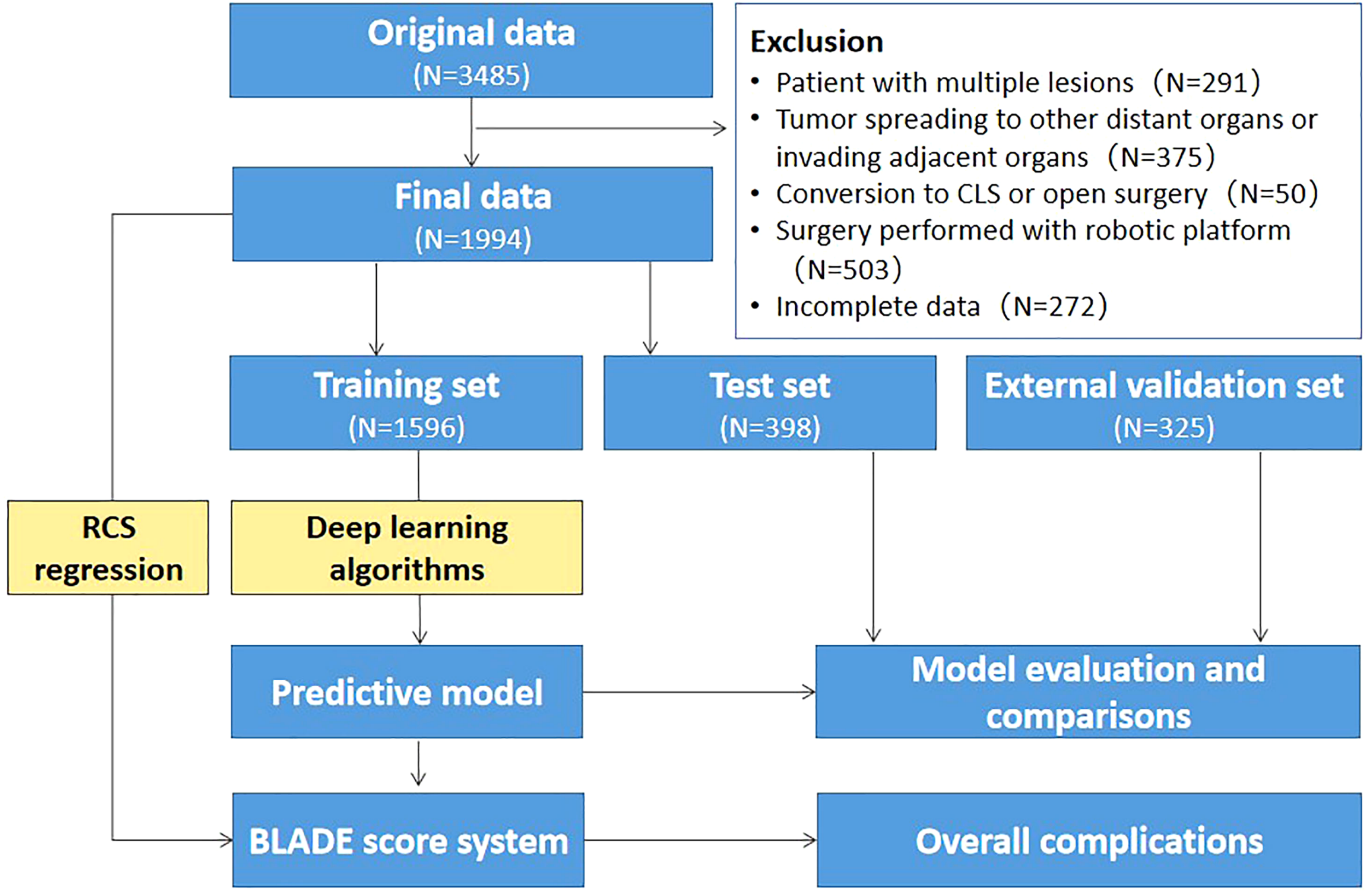
Flowchart illustrating patient selection and the overall data analysis procedures. RCS, restricted cubic spline; BLADE, blood loss and duration of excision; LR, logistic regression; CLS, conventional laparoscopic surgery.

A total of 3,485 RC patients treated with tLAR between August 2008 and July 2021 were collected from the China national database of tLAR and NOSE for colorectal cancer. The data were collected by a secure online platform (http://chinanoses.yiducloud.com.cn) and stored in a uniform format. This study was reviewed and approved by the institutional review board of China National Cancer Center and was exempt from patient consent given the retrospective nature of the study. All included patients were pathologically diagnosed with adenocarcinoma located within 15 cm from the anal verge. The exclusion criteria for tLAR were as follows: patient with multiple lesions, tumor spreading to other distant organs or invading adjacent organs, the patient underwent conversion to conventional laparoscopic surgery or open surgery, surgery performed with a robotic platform, and patient with incomplete data. The flowchart is presented in **Figure 1**.

### Variable selection

The clinical records of RC patients were extracted with the following information for analysis: patient characteristics [gender, age at diagnosis, body mass index (BMI), and comorbidity], tumor characteristics [distance from lower edge of tumor to anus, tumor size, American Joint Committee on Cancer (AJCC) TNM stage, preoperative serum carcinoembryonic antigen (CEA), preoperative serum carbohydrate antigen 19-9 (CA19-9), and receipt of neoadjuvant chemoradiotherapy], surgical information (estimated blood loss and surgical time), and 30-day postoperative complications (anastomotic leakage, anastomotic bleeding, anastomotic stenosis, intraabdominal bleeding, intraabdominal abscess, rectovaginal fistula, intestinal obstruction, wound complications, pulmonary disease, urinary disease, and others).

### Surgical procedures of tLAR

All surgical procedures of tLAR were performed by experienced colorectal surgeons for laparoscopic surgery. The tLAR was performed as described previously (23). Briefly, the main surgical procedures of tLAR were as follows: a) anterior resection was performed following the principle total mesorectal excision (TME), b) digestive tract reconstruction included low colorectal end-to-end anastomosis or coloanal end-to-end anastomosis, and c) the rectal specimen was finally extracted transanally or transvaginally. In brief, all procedures of anterior resection and digestive tract reconstruction were performed intraabdominally.

### Development of BLADE scoring system

The surgical difficulty grading of the BLADE scoring system was built by two surgical variables including duration of surgery and estimated blood loss. Operative time was defined as the time from skin incision to final cutaneous closure. Anesthesiologists carefully evaluated blood loss during the operation and recorded it at the end of the operation. Restricted cubic spline (RCS) regression plots were performed to examine the full-range associations between the duration of surgery and the estimated blood loss with the odd ratios (ORs) for overall complication within 30 days to ascertain the optimal cutoff point to classify the operative time and total intraoperative blood loss into binary variables with a certain degree of objectivity. Each of the two intraoperative factors was assigned 1 point when it was at or above the threshold value. Therefore, the BLADE score ranged from 0 to 2, and patients scoring 0, 1, and 2 were classified as low, middle, and high difficulty of tLAR, respectively.

### Establishment of the preoperative model to predict surgical difficulty

Of included patients from the national database, 80% (n = 1,596) were randomly selected for the training set, and the remaining 20% (n = 398) were used as the test set. Furthermore, 325 RC patients who underwent tLAR between January 2015 and August 2018 at Cancer Hospital Chinese Academy of Medical Sciences and the Second Affiliated Hospital of Harbin Medical University were identified as the external validation set according to the inclusion and exclusion criteria. The preoperative models for the BLADE score were developed based on the training cohort by using machine learning algorithms and then were tested in both the test set and the external validation cohort. Nine preoperative variables associated with surgical difficulty were obtained, including gender, age at diagnosis, BMI, history of previous diseases, receipt of neoadjuvant chemoradiotherapy, tumor location, tumor size, AJCC T stage, and AJCC N stage. The algorithms included logistic regression (LR), k-nearest neighbor (KNN), support vector machine (SVM), artificial neural network (ANN), generalized boosting machines (GBMs), and random forest (RF). The details of each model are described in **Supplementary Table 1**. We calculated the area under the receiver operating characteristic curve (AUC) as our primary performance metric to assess the discrimination of the machine learning algorithm.

### Statistical analysis

The data were presented as the mean with standard deviation (SD) for continuous variables and proportions (%) for categorical variables. The association between surgical difficulty and overall postoperative complications was evaluated through uni- and multivariate binary logistic regression analyses by calculating ORs and 95% confidence intervals (CIs). The variables with a *p-*value of less than 0.05 after univariate analysis were included in the multivariate analysis. Statistically significant results were defined as *p* < 0.05, and all *p*-values were two-sided. Data analyses were performed using IBM SPSS Statistics version 23.0 (IBM Corp.) and R software version 3.5.3 (R Project for Statistical Computing). The study was reported in line with the STROCSS criteria (24).

## Results

### Patient characteristics and surgical outcomes

A total of 1,994 patients who underwent tLAR were identified, including 1,030 (51.7%) male and 964 (48.3%) female patients, with a median age of 60.20 (SD = 11.43) years and a mean BMI of 22.84 kg/m^2^ (SD = 2.96). A total of 617 (30.9%) patients had comorbidities, and 101 patients (5.1%) had received neoadjuvant therapy. The mean duration of surgery was 188.59 (range 75–669) min, and the mean intraoperative blood loss was 75.85 (range 40–600) ml. The overall complication rate was 17.9% (356 of 1,994 cases). The details of patient information are shown in **Table 1**.

**Table 1:** Demographic and tumor characteristics of patients undergoing tLAR for RC.

Characteristics	Derivation set	Training set	Test set	External validation set
Gender, n (%)				
Male	1,030 (51.7)	835 (52.3)	195 (49.0)	203 (49.0)
Female	964 (48.3)	761 (47.7)	203 (51.0)	211 (51.0)
Age at diagnosis, mean (SD), years	60.20 (11.43)	60.08 (11.59)	60.67 (10.73)	61.54 (10.67)
Age at diagnosis, n (%), years				
<60	885 (44.4)	717 (44.9)	168 (42.2)	157 (37.9)
≥60	1,109 (55.6)	879 (55.1)	230 (57.8)	257 (62.1)
BMI, mean (SD), kg/m^2^	22.84 (2.96)	22.836 (2.98)	22.87 (2.91)	22.49 (2.90)
BMI, n (%), kg/m^2^				
<18.5	113 (5.7)	95 (6.0)	18 (4.5)	25 (6.0)
≥18.5, <25	1,437 (72.1)	1,144 (71.7)	293 (73.6)	308 (74.4)
≥25, <30	420 (21.1)	337 (21.1)	83 (20.9)	74 (17.9)
≥30	24 (1.2)	20 (1.3)	4 (1.0)	7 (1.7)
Comorbidity, n (%)				
No	1,377 (69.1)	1,107 (69.4)	270 (67.8)	86 (20.8)
Yes	617 (30.9)	489 (30.6)	128 (32.2)	328 (79.2)
Neoadjuvant chemoradiotherapy, n (%)				
No	1,893 (94.9)	1,517 (95.1)	376 (94.5)	403 (97.3)
Yes	101 (5.1)	79 (4.9)	22 (5.5)	11 (2.7)
Tumor location, n (%), cm				
<5	422 (21.2)	327 (20.5)	95 (23.9)	93 (28.6)
≥5, <10	695 (34.9)	570 (35.7)	125 (31.4)	144 (44.3)
≥10	877 (44.0)	699 (43.8)	178 (44.7)	88 (27.1)
Tumor size, mean (SD), cm	3.53 (1.39)	3.559 (1.42)	3.43 (1.24)	3.37 (1.14)
Tumor size, n (%), cm				
<5	1,681 (84.3)	1,339 (83.9)	342 (85.9)	360 (87.0)
≥5	313 (15.7)	257 (16.1)	56 (14.1)	54 (13.0)
T stage, n (%)				
T0–T2	737 (37.0)	573 (35.9)	164 (41.2)	348 (87.0)
T3–T4	1,257 (63.0)	1,023 (64.1)	234 (58.8)	66 (15.9)
N stage, n (%)				
N0	1,313 (65.8)	1,049 (65.7)	264 (66.3)	255 (61.6)
N1–2	681 (34.2)	547 (34.3)	134 (33.7)	159 (38.4)
CEA, n (%)				
Normal	1,327 (66.5)	1,056 (66.2)	271 (68.1)	228 (55.1)
Elevated	667 (33.5)	540 (33.8)	127 (31.9)	186 (44.9)
CA19-9, n(%)				
Normal	1,529 (76.7)	1,212 (75.9)	317 (79.6)	257 (62.1)
Elevated	465 (23.3)	384 (24.1)	81 (20.4)	157 (37.9)
Duration of surgery, mean (SD), min	188.59 (70.36)	188.48 (70.59)	189.06 (69.50)	157.32 (56.06)
Estimated intraoperative blood loss, mean (SD), ml	75.85 (47.37)	76.06 (48.04)	75.05 (44.61)	70.08 (51.31)
Postoperative complication, n (%)				
No	1,638 (82.1)	1,314 (82.3)	324 (81.4)	378 (91.3)
Yes	356 (17.9)	282 (17.7)	74 (18.6)	36 (8.7)

RC, rectal cancer; BMI, body mass index; SD, standard deviation; CEA, carcinoembryonic antigen; CA19-9, carbohydrate antigen 19-9; tLAR, total laparoscopic anterior resection.

### Development of BLADE scoring system

**Figure 2:**
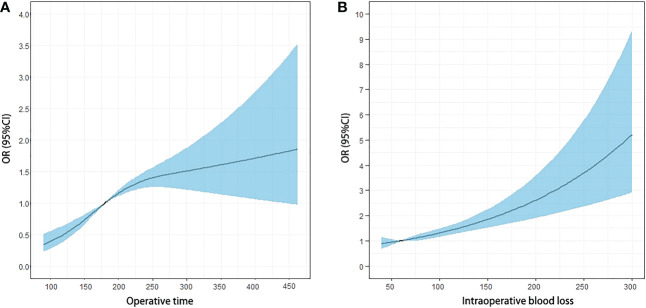
Odds ratio (OR) curves of the duration of surgery and the estimated blood loss for postoperative complication risk with spline curve analysis. **(A)** The OR continuously increased with the increase of the duration of surgery, and the plateau phase of the curve was detected around 165 and 281 min. The plateau phase continued until 281 min, and the OR increased again with the increase in the duration of surgery. **(B)** The estimated blood loss was associated with the OR of complication in a linear profile.

The effects of the duration of surgery and the estimated intraoperative blood loss on the ORs and 95% CI of overall complication were present using spline curve analysis. For the duration of surgery, the ORs continuously increased with an increase in the duration of surgery, and the slight plateau phase of the curve was detected between approximately 165 and 281 min (non-linearity *p*-values were 0.001) (**Figure 2A**). Increasing the duration of surgery at <165 and >281 min was associated with a rapid increase in the risk of overall complications after surgery. We then defined the duration of surgery performed ≥165 min as long duration (1 point) and the duration of surgery performed <165 min as short duration (0 point). The estimated intraoperative blood loss was associated with complications in a linear profile (non-linearity *p*-values were 0.911) (**Figure 2B**). Thus, we defined blood loss >60 ml (OR, 1.01; 95% CI, 0.996–1.015) as a large amount of bleeding (1 point) and ≤60 ml as a small amount of bleeding (0 point). Based on this new scoring system, 1,994 patients were scored retrospectively; 517 (25.9%), 989 (49.6%), and 488 patients (24.5%) were defined as low-, middle-, and high-difficulty groups, respectively (**Table 2**).

**Table 2:** Patient proportion of surgical difficulty for tLAR according to BLADE scoring system.

	Derivation set	Training set	Test set	External validation set
Operation time score, n (%)				
0	863 (43.3)	690 (43.2)	173 (43.5)	288 (69.6)
1	1,131 (56.7)	906 (56.8)	225 (56.5)	126 (30.4)
Operative blood loss score, n (%)				
0	1,158 (58.1)	928 (58.1)	230 (57.8)	233 (56.3)
1	836 (41.9)	668 (41.9)	168 (42.2)	181 (43.7)
BLADE score, n (%)				
0	517 (25.9)	406 (25.4)	111 (27.9)	166 (40.1)
1	989 (49.6)	807 (50.6)	182 (45.7)	189 (45.7)
2	488 (24.5)	383 (24.0)	105 (26.4)	59 (14.3)

tLAR, total laparoscopic anterior resection; BLADE, blood loss and duration of excision.

### Effect of BLADE score on postoperative complication

The multivariate logistic analysis was used to identify the association between the BLADE score and postoperative complication. In the derivation set, we found that male patients (OR, 1.438; 95% CI, 1.132–1.826, *p* = 0.003), patients with comorbidity (OR, 1.774; 95% CI, 1.390–2.265, *p* = 0.000), lower tumor location (OR, 2.183; 95% CI, 1.615–2.953, *p* = 0.000), and the BLADE scoring system (middle-difficulty, OR, 1.408; 95% CI, 1.013–1.955, *p* = 0.042; high-difficulty, OR, 2.423; 95% CI, 1.702–3.450, *p* = 0.000) were considered as the independent risk factors to postoperative complication for patients treated with tLAR. Similar findings of the association between the surgical difficulty of the BLADE score and complication were also presented in the external validation set (**Table 3**). The results above suggested that patients with higher difficulty levels were associated with a higher risk of complication after tLAR.

**Table 3:** Univariate and multivariate logistic regression analyses of postoperative complication.

	Derivation set				External validation set			
	Univariate analysis		Multivariate analysis		Univariate analysis		Multivariate analysis	
	OR (95% CI)	*p*	OR (95% CI)	*p*	OR (95% CI)	*p*	OR (95% CI)	*p*
Gender								
Male	1.435 (1.137–1.810)	0.002	1.438 (1.132–1.826)	0.003	1.710 (0.849–3.444)	0.133		
Female	Ref.		Ref.		Ref.			
Age, years								
<60	Ref.				Ref.			
≥60	1.028 (0.816–1.295)	0.814			0.657 (0.331–1.307)	0.231		
BMI, kg/m^2^								
<18.5	Ref.				Ref.			
≥18.5, <25	0.671 (0.426–1.056)	0.084			2.306 (0.300–17.717)	0.422		
≥25, <30	0.626 (0.378–1.036)	0.068			2.507 (0.293–21.452)	0.401		
≥30	2.275 (0.907–5.706)	0.080			4.000 (0.217–73.618)	0.351		
Comorbidity								
No	Ref.		Ref.		Ref.		Ref.	
Yes	1.880 (1.485–2.380)	0.000	1.774 (1.390–2.265)	0.000	0.284 (0.140–0.576)	0.000	0.487 (0.218–1.087)	0.079
Neoadjuvant therapy								
No	Ref.				Ref.			
Yes	0.860 (0.498–1.486)	0.588			1.051 (0.131–8.455)	0.962		
Tumor location, cm								
<5	2.590 (1.947–3.447)	0.000	2.183 (1.615–2.953)	0.000	2.225 (0.861–5.751)	0.099		
≥5, <10	1.263 (0.956–1.668)	0.100	1.178 (0.885–1.567)	0.261	0.864 (0.316–2.358)	0.775		
≥10	Ref.		Ref.		Ref.			
Tumor size, cm								
<5	Ref.				Ref.			
≥5	1.312 (0.973–1.768)	0.075			2.062 (0.887–4.796)	0.093		
T stage								
T0–T2	Ref.		Ref		Ref.		Ref.	
T3–T4	0.789 (0.625–0.997)	0.047	0.863 (0.677–1.101)	0.248	5.248 (2.550–10.799)	0.000	2.593 (1.135–5.922)	0.024
N stage								
N0	Ref.				Ref.		Ref.	
N1–N2	0.976 (0.766–1.244)	0.845			2.149 (1.078–4.284)	0.030	2.295 (1.061–4.966)	0.035
BLADE scoring system								
0	Ref.		Ref.		Ref.		Ref.	
1	1.581 (1.145–2.183)	0.005	1.408 (1.013–1.955)	0.042	3.389 (1.230–9.343)	0.018	3.221 (1.144–9.072)	0.027
2	3.150 (2.245–4.420)	0.000	2.423 (1.702–3.450)	0.000	9.100 (3.084–26.856)	0.000	6.261 (1.880–0.851)	0.003

BMI, body mass index; BLADE, blood loss and duration of excision.

### Establishment of the preoperative model to predict surgical difficulty

**Figure 3:**
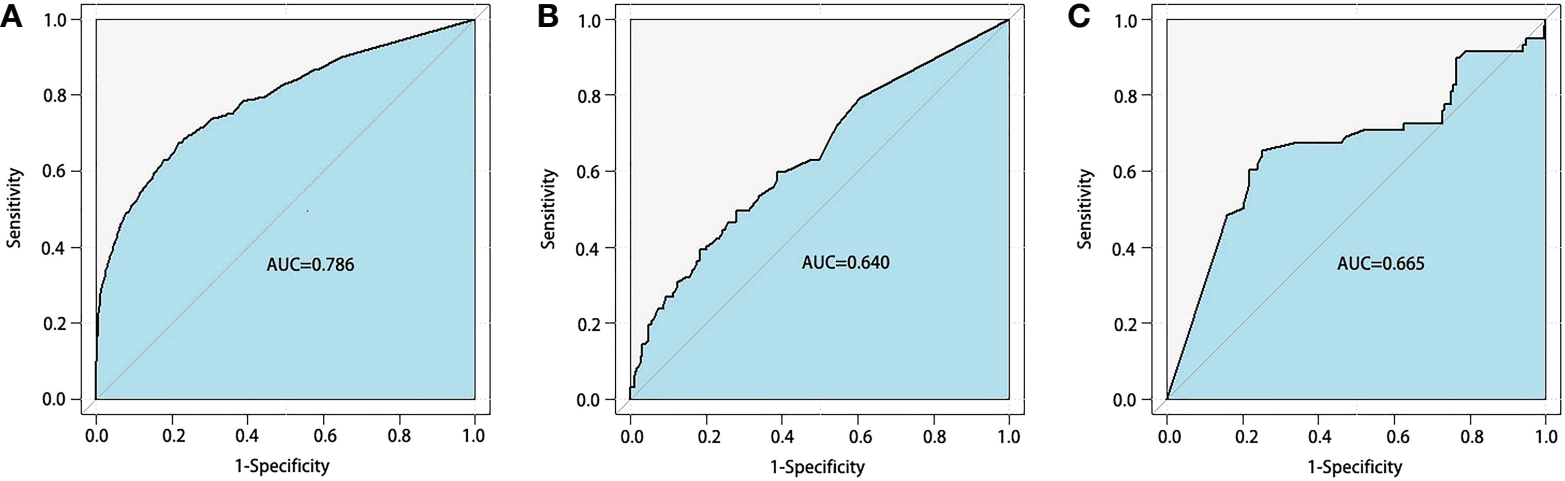
Receiver operating characteristics curves of random forest model for BLADE scoring system. The areas under the curve (AUCs) were 0.786 in the training set **(A)** 0.640 in the test set **(B)** and 0.665 in the external validation set **(C)** BLADE, blood loss and duration of excision.

In order to identify the high-difficulty group, we combined patients in the low-difficulty group and patients in the middle-difficulty into one group. For logistic regression, we found that tumor location, comorbidity, and neoadjuvant therapy were considered predictors for the surgical difficulty of tLAR for RC patients (**Supplementary Table 2**). Moreover, we found that the AUC of the RF algorithm (0.786 in the training set; 0.640 in the test set; 0.665 in the external validation set, **Figure 3**) was significantly better than that of other models (**Supplementary Table 1**).

## Discussion

Individualized treatment has been gradually emphasized in current clinical practice, and a useful and easy scoring system of surgical difficulty could help to identify patients with a high risk of having postoperative complications and patients with poor prognoses. Here, our study is the first report to develop an easy-to-use BLADE scoring system to evaluate the surgical difficulty for tLAR and validate the performance in an independent external cohort to evaluate the ability of true replication, which could reflect the generalizability of this scoring system in the clinical setting. Then, we used preoperative variables to establish the predictive model for the BLADE score based on machine learning algorithms.

The assessment of surgical difficulty is challenged by multiple factors that depend on the surgeon’s experiences, the cooperation of the surgical team, and the surgical platform (25). Therefore, the variable selection in the grading system of surgical difficulty is sometimes debatable and subjective. Escal et al. recently developed a grading system to evaluate the surgical difficulty of TME for locally advanced RC, including six intraoperative and postoperative variables, including conversion to laparotomy, blood loss, duration of surgery, use of transanal dissection (the transanal approach is required to complete TME in difficult surgical case), postoperative complications, and length of hospital stay. Then, a grading system was established based on these variables to classify the RC patients as at low-risk or high-risk of surgical difficulties (26). Based on this score grading system, Chen et al. (27), Yamamoto et al. (28), and de’Angelis et al. (29) made small modifications of variables to this system according to their own needs and then established the risk models to predict the surgical difficulty. The establishment of the above grading systems was based on intraoperative and postoperative variables, which indicated that both an unsuccessful resection and an extended postoperative course were related to surgical difficulty (26). However, we believe that the inclusion of postoperative variables into the scoring system should be cautiously considered for two reasons. First, the postoperative outcome, such as the length of hospital stay, is affected by a variety of uncontrollable factors, which makes it impossible to discern the association between the postoperative outcome and the surgical difficulty. Second, the above studies did not calculate the correlation between intraoperative variables and postoperative outcomes, leading to the inability to ensure that the variable selection met the statistical requirements of model establishment. Therefore, it is scientific and reasonable to establish a surgical difficulty evaluation system only based on intraoperative variables, which could objectively reflect the degree of difficulty in the surgical process. The score grading systems based on intraoperative variables have been established and validated in various types of surgery (30–36). In our study, we established a simple scoring system based on the intraoperative parameters of blood loss and duration of surgery, which was validated as having close associations with postoperative complications. In general, studies selected a median or alternative value as the cutoff value to divide patients into different groups, which weakens its clinical guiding significance. The results of the present study showed that although ORs continuously increased with an increase in the duration of surgery or blood loss, the RCS model (37) demonstrated a non-linear association between continuous operative time and outcome. Therefore, the optimal cutoff value should be 165 min, which maximizes the differences in ORs since the risk of postoperative complications increased at different rates before and after 165 min of surgical time. In contrast, the association between intraoperative blood loss and ORs of complications after surgery was linear. Blood loss <60 ml was the protective factor against complication, and when blood loss >60 ml, the ORs of complication were greater than 1. Therefore, we chose 60 ml as the optimal cutoff.

In addition, a previous study has established a surgical difficulty scoring system for TME surgery based on preoperative variables. Baek et al. have established a scoring system to assess the surgical difficulty of robotic surgery for RC according to MRI-based pelvimetry, including large tumor size, narrow intertuberous distance, shallow sacral angle, and long sacral length (38). Then, they categorized patients into three risk groups based on four risk factors: easy group (no risk factor), moderate group (one to two risk factors), and difficult group (three to four risk factors). There are many controversies in using preoperative variables to evaluate a surgical difficulty, but they should be considered as predictors of surgical difficulty to assist surgical decision-making. Several studies have identified many variables to predict the surgical difficulty of rectal resections. Gender, BMI, tumor location, tumor size, comorbidity, pelvic anatomical structure, neoadjuvant therapy, and surgeon experiences were identified as predictive factors for the duration of surgery, conversion to open surgery, and postoperative complications (26, 39–41). Similar to the results of previous studies, we found that tumor location, comorbidity, and neoadjuvant therapy were considered predictors for the surgical difficulty of tLAR for RC patients.

In light of recent developments in machine learning and the accessibility of computing power, the application of the technique in the data mining and model development field has yielded promising results (42). Currently, most of the predictive tools are presented with limited clinical applicability, poor predictive ability, and lack of external validation (28, 43, 44) since they are developed according to the variables’ interaction in a linear and additive manner (45), but the surgical difficulty is multi-factorial, and the interaction between surgical difficulty and influencing factors cannot be completely linear. Machine learning algorithms could effectively overcome the shortcomings of traditional methods, which can be used as a more accurate and non-linear tool to predict the outcomes of patients (46, 47). They can easily incorporate a large number of variables, as all calculations are performed using a computer to offer insights into latent interactions between numerous input features and output results to achieve output prediction (48). In the field of prediction, machine learning techniques are increasingly used in various areas including outcome prediction (49), but not in surgical difficulty prediction. The approach of machine learning is independent of complex interactions, which could lead to higher prediction accuracy. Therefore, we developed models using machine learning techniques to predict the difficulty of tLAR. This study demonstrated that the use of machine learning models can accurately predict the difficulty of tLAR. The results showed that the RF model presented a better performance for the prediction of the difficulty of tLAR than the other models. We also externally validated the models in a large cohort in which patient characteristics were broadly similar to the original derivation dataset, thus enabling a head-to-head comparison of the models. Notably, what is different from usual was that the predictive model performed better in the external validation dataset than in the internal validation cohort, which indicated that our predictive tool had the ability to identify surgical difficulty grades.

There are several limitations in this study. First, because a retrospective analysis was used, there are relatively heterogeneous data regarding the determination of tumor location based on different imaging protocols, surgical technique selection of tLAR, and the skills and experiences of surgeons. Second, pelvimetry in pelvic MRI plays an important role in determining the surgical difficulties of anterior resection. However, the information with regard to MRI was missing in the database, which could not be analyzed in this study. Third, the surgeon’s experiences have been considered a key influencing factor for surgical difficulty, but we cannot calculate the influence of the surgeon’s experience on this scoring system due to the lack of relevant information in this database. Fourth, the establishment and evaluation of the surgical difficulty grading system in anterior resection varied obviously between studies, which are unavailable for the comparison of our grading score with the others. Fifth, the subjectivity of the definition of surgical difficulty remains largely unaddressed, which likely leads to potential bias and makes the relationship between surgical difficulty and clinical outcomes difficult to explain. Despite the retrospective nature and limitations in the present study, the advantages of this study include that the surgical difficulty score grading of tLAR is established based on a large sample size of RC patients, and further investigations of the current scoring system should be performed with internal cohort and independent external cohort to validate the outcomes.

## Conclusions

The easy-to-use BLADE score appears to be effective in predicting the short-term outcome for patients who are candidates to receive tLAR, convenient in making surgical plans for RC patients, and significant in promoting more studies for tLAR in both multicenter studies and randomized clinical trials in the near future.

## Data availability statement

The original contributions presented in the study are included in the article/**Supplementary Material**. Further inquiries can be directed to the corresponding author.

## Ethics statement

The studies involving human participants were reviewed and approved by the institutional review board of China National Cancer Center. Written informed consent for participation was not required for this study in accordance with the national legislation and the institutional requirements.

## Author contributions

JFL: methodology, software, data curation, and writing—original draft preparation. XG: conceptualization, writing—original draft preparation, and data curation. RW: software. YFY: data curation. ERL: software. ZXZ: visualization. HPC: investigation. ZL: resources. ZJ: resources. XSW: supervision. All authors contributed to the article and approved the submitted version.
