# Supplementary material for: Development of artificial blood loss and duration of excision score to evaluate surgical difficulty of total laparoscopic anterior resection in rectal cancer
Source: Front Oncol. 2023 Mar 7;13:1067414. doi: 10.3389/fonc.2023.1067414 (PMC10028132; doi:10.3389/fonc.2023.1067414)
Supplement: Supplementary file 1 [file DataSheet_1.pdf]

Supplementary Material

Supplementary Table 1. The receiver operating characteristic curve of prediction models under consideration.

Model	Training set	Test set	External validation set
Logistic regression (LR)	0.675	0.618	0.306
K-nearest neighbor (KNN)	0.722	0.556	0.447
Support vector machine (SVM)	0.673	0.546	0.494
Artificial neural network (ANN)	0.717	0.556	0.623
Gradient boosting machine (GBM)	0.697	0.578	0.660
Random forest (RF)	0.782	0.640	0.665

Note: The detailed explanations for predictive model were as follows:

1. Logistic regression (LR): LR, which is an extension of ordinary regression, helps to find the probability that a new instance belongs to a certain class. It can model only a dichotomous variable which usually represents the occurrence or non-occurrence of the event.

2. K-nearest neighbor (KNN): The aim of KNN is to identify k-nearest training example in the feature space and the classification is decided by a plurality vote of its neighbors. The k-value, which is the number of the nearest neighbor, is important in determining the model efficiency. We set the distance as 1. The remaining parameters were set to default values.

3. Support vector machine (SVM): Firstly, SVM maps each data into an n-dimensional feature space. Then, it identifies the hyperplane that separates and classifies the data into distinct classes in a higher dimensional space, maximizing the marginal distance for both classes and minimizing the classification errors. Indeed, assuming that training data has been labeled as belonging to one of two sets, SVM is a discriminative classifier. In this study, the kernel we used is radial basis to solve the non-linear problem. The cost of contraints violation value was set to 2 and the gamma value was set to 0.8. The remaining parameters were set to default values.

4. Artificial neural network (ANN): ANN comprises layers of interconnected artificial neurons. An artificial neuron is designed based on the biological neuron itself and receives multiple inputs multiplied by weights (adjust signal strengths of communication) and outputs the sum of the inputs to another node for subsequent processing according to the interconnection. Nodes are grouped into a matrix called layer. Apart from the input and output layer, there can be multiple hidden layers. In this

study, we used 3 hidden layers and the parameter for weight decay was 0.1. The remaining parameters were set to default values.

5. Generalized boosting machines (GBM): GBM uses many smaller, weaker models and brings them together into a final summed prediction. In each iteration, a new weak model is trained with respect to the whole ensemble learned up to that new model. These new models are built to be maximally correlated with the negative gradient of the loss function that is also associated with the ensemble as a whole. In this approach, a performance function is placed on the GBM in order to find the point at which adding more iterations becomes negligible in benefit. At this point, the ensemble sums all of the predictions into a final overall prediction. In this study, the number of trees and the minimum observations per node were set at 500 and 50, respectively. The remaining parameters were set to default values.

6. Random forest (RF): Decision tree (DT) models the decision logic into a tree-like structure and consists of multiple levels of nodes and classification algorithm. All internal nodes represent tests on input variables or attributes. Depending on the test outcome, the classification algorithm branches towards the appropriate child node until it reaches the leaf node, which correspond to the decision outcomes. RF consists of a multitude of independent DTs. The different DTs of an RF are trained using the random subset of the training set as a different part of input vector and gives a classification outcome. The sum of the decisions made by the DTs is used for the final classification. RF is to find the highest rank among all tree classifiers. In the study, after optimization, we used 500 decision trees, nine variables randomly sampled as candidates at each split. The remaining parameters were set to default values.

Supplementary Table 2. Univariate and multivariate logistic regression analysis of BLADE score system

	**Univariate analysis**		**Multivariate analysis**	
	**OR (95% CI)**	***p***	**Adjusted OR (95% CI)**	***p***
**Gender**				
Male	0.928(0.737-1.168)	0.524		
Female	Ref.			
**Age, years**				
<60	Ref.			
≥60	0.889(0.706-1.120)	0.320		
**BMI, kg/m^2**				
<18.5	Ref.			
≥18.5, <25	1.268(0.753-2.136)	0.372		
≥25, <30	1.183(0.674-2.075)	0.558		
≥30	1.692(0.574-4.985)	0.340		
**Comorbidity**				
No	Ref.		Ref.	
Yes	1.831(1.441-2.326)	0.000	1.900(1.483-2.434)	0.000
**Neoadjuvant therapy**				
No	Ref.		Ref.	
Yes	3.930(2.547-6.063)	0.000	2.846(1.816-4.459)	0.000
**Tumor location, cm**				

<5	3.144(2.328-4.246)	0.000	2.945(2.159-4.018)	0.000
≥5, <10	1.844(1.399-2.431)	0.000	1.760(1.326-2.336)	0.000
≥10	Ref.		Ref.	
**Tumor size, cm**				
＜5	Ref.			
≥5	1.325(0.984-1.785)	0.064		
**T**				
T0-T2	Ref.			
T3-T4	0.790(0.624-1.001)	0.051		
**N**				
N0	Ref.			
N1-N2	0.884(0.692-1.129)	0.323		
